# ﻿*Didymodonchangbaiensis* (Pottiaceae, Musci), a new species from Changbai Mountain, China and its phylogenetic position based on molecular data

**DOI:** 10.3897/phytokeys.221.96661

**Published:** 2023-03-13

**Authors:** Ting-Ting Wu, Chao Feng, Tao Bian, Guo-Li Zhang, Jin Kou, Hong-Xing Xiao

**Affiliations:** 1 School of Life Sciences, Jilin Provincial Key Laboratory of Chemistry and Biology of Changbai Mountain Natural Drugs, Northeast Normal University, Changchun 130024, China Northeast Normal University Changchun China; 2 College of Grassland, Resources and Environment, Key Laboratory of Grassland Resources, Ministry of Education P.R. of China, Key Laboratory of Forage Cultivation, Processing and High Efficient Utilization of Ministry of Agriculture, Inner Mongolia Agricultural University, Hohhot 010011, China Inner Mongolia Agricultural University Hohhot China; 3 Faculty of Life Science, Tangshan Normal University, Tangshan 063000, China Tangshan Normal University Tangshan China

**Keywords:** Asia, northeast China, phylogenetic analysis, taxonomy

## Abstract

Changbai Mountain, located in northeast China, is one of the areas with the most complete natural ecosystem preservation in China. A new species, *Didymodonchangbaiensis* C.Feng, J.Kou, H.-X. Xiao & T.-T.Wu from north slope of Changbai Mountain in Jilin Province of China is described and illustrated. It is characterised by ovate or ovate-lanceolate leaves that are appressed when dry, acute leaf apex, lamina red or reddish-orange with KOH, unistratose lamina throughout, plane and unistratose leaf margins, percurrent costa with one layer of guide cells and without ventral stereids, upper and middle laminal cells with elliptical papillae over the transverse walls between two immediately adjacent cells and basal laminal cells not differentiated from the median cells. Our morphological analyses and molecular results, based on DNA sequences of ITS, *rps*4 and *trn*M-*trn*V, conﬁrm that *D.changbaiensis* is revealed to be sister to *D.daqingii* J. Kou, R.H. Zander & C. Feng. This new species is compared with similar species and its phylogenetic position and ecology are discussed.

## ﻿Introduction

Changbai Mountain National Reserve is located at the junction of three counties: Antu County, Fusong County and Changbai County in the southeast of Jilin Province, with a total area of 196,000 ha^2^. It is one of the earliest national nature reserves established in China. The vertical height difference of the Nature Reserve is nearly 2000 m and the altitude is between 720 and 2691 m (Jia et al. 2020). From bottom to top, the landform can be clearly divided into three annular zones: lava platform, lava plateau and volcanic cone. The protected area belongs to the temperate humid monsoon climate, which is generally characterised by long and cold winters, short and cool summers, strong and dry winds in spring and foggy and cool autumns. The plants in the area belong to the flora of Changbai Mountain and the vegetation changes vertically with the altitude gradient. The vegetation in this area has obvious vertical zonal distribution due to the influence of climate and it is generally divided into four vertical vegetation zones. From the foot of the mountain to the top of the mountain are generally: broad-leaved Korean pine forest belt (below 1100 m), spruce fir forest belt (1100–1800 m), Yuehua forest belt (1800–2100 m) and alpine tundra belt (greater than 2100 m) (Ping et al. 2016). Due to the diverse characteristics of topography, climate and ecosystem, the area is rich in biodiversity resources. There are nine species of amphibians, 12 species of reptiles, 24 species of fish, 56 species of mammals, 230 species of birds and 1255 species of insects in the Reserve, respectively (Tang et al. 2011). In addition, there are 430 species of fungi, 200 species of lichens, 311 species of bryophyte, 78 species of ferns, 11 species of gymnosperms and at least 1325 species of neutrophils (Zhang et al. 2016).

Although the bryophyte flora of Changbai Mountain has been well studied, most of these studies were nearly twenty years ago (e.g. Koponen et al. 1983; Cao and Guo 2000; Cao et al. 2002; Guo et al. 2002). In the background of the recent revolution of genus *Didymodon* (Zander 2013; Zander 2019) which was split into seven smaller genera: *Aithobryum* R.H. Zander, *Didymodon* s. str., *Exobryum* R.H. Zander, *Fuscobryum* R.H. Zander, *Geheebia* Schimp., *Trichostomopsis* Card. and *Vinealobryum* R.H. Zander, based on macro-evolutionary analysis and the dissilient genus concept applied (Zander 2013; Zander 2019), a re-evaluation of *Didymodon* in China is being conducted by the authors (e.g. Feng et al. 2022a; Feng et al. 2022b). During a recent excursion in Changbai Mountain, many specimens have been collected. Amongst them, two samples of *Didymodon* s. lat. from stony habitats are different from species previously reported in the area (Li et al. 2001). They mostly resemble *Didymodontibeticus* J.Kou, X.-M.Shao & C.Feng. To clarify their taxonomic identity, we conducted phylogenetic analysis and confirm that these samples belong to the genus *Didymodon* s. str. (Zander 2013), but do not match with any species known in the genus. Here, we describe this unknown moss as a new species.

## ﻿Materials and methods

### ﻿Morphological observations

Over 3000 specimens of the genus *Didymodon* s. lat. were examined during our revision of Pottiaceae in China. More than 50 field investigations were conducted in past years and the specimens included in this study were housed in the Herbaria at IFP, KUN and NMAC. Microscopic examinations and measurements were taken with a ZEISS Primo Star light microscope and photomicrographs were obtained with a Canon EOS 70D camera, mounted on this microscope. Specimens were examined in 2% potassium hydroxide (KOH). Three plants were dissected from each collection and, for each shoot, every possible structure from the gametophyte had to be examined and a record kept of what was found for each individual species. Specific morphological and anatomical features of taxonomic importance were assessed mainly following Zander (1993). Leaves were always taken from the upper and middle parts of the stem and cross-sections were made in the middle part of the stem. Measurements of leaf width were taken at the base, mid-leaf and upper part. Cross-sections were made at mid-leaf.

### ﻿Phylogenetic analyses

To test the phylogenetic position of the new species, one specimen (234) collected from Changbai Mountain was sampled. Another species (188) that was discovered nearby the new species was also sampled. We employed one nuclear (ITS) and two chloroplast markers (*rps*4 and *trn*M-*trn*V), which had been used successfully in previous phylogenetic studies in *Didymodon* s. lat. and enabled the re-use of earlier results and easier interpretation of new data (Zhang et al. 2022). Phylogenetic trees are created and shown separately. The complete list with sample names and GenBank accession numbers is presented in Tables 1 and 2. DNA extraction, PCR ampliﬁcation and sequencing procedure followed the protocols described by Wang et al. (2010).

**Table 1.** New sequences used in this study, including taxa vouchers information and GenBank accession numbers.

Species	Voucher information	ITS	*rps*4	*trn*M-*trn*V
234 (new species)	China, Changbai Mountain, Jin Kou 20200902234	OP641837	OP641840	OP656316
188 (unknown species)	China, Changbai Mountain, Jin Kou 20200902188	OP641838	OP641839	OP656315

**Table 2.** Sequences from GenBank used in this study, including taxa and GenBank accession numbers.

Species	ITS	*rps4*	*trnM-trnV*
*Acaulontriquetrum*	MW398556		
*Aloinarigida*	MW398549		
*Aloinellaandina*	MW398550		
*Andinellachurchilliana*	MW398720		
*Andinellacoquimbensis*	MW398711		
*Andinellaelata*	MW398708		
*Andinellagranulosa*	MW398714		
*Andinellalimensis*	MW398710		
*Andinellaoedocostata*	MW398733		
*Andinellapruinosa*	MW398726		
*Barbulaunguiculata*	MW398553	HM147777	JQ890366
*Bryoerythrophyllumrecurvirostrum*	MW398547	JQ890468	JQ890407
*Bryoerythrophyllumrubrum*	MW398548		
*Chenialeptophylla*	MW398561		
*Cinclidotusriparius*	MW398554		
*Crossidiumsquamiferum*	MW398558		
*Didymodonacutus*	AY437111	KP307551	KP307667
*Didymodonalpinus*	MW398606		
*Didymodonandreaeoides*	MW398768		
*Didymodonanserinocapitatus*	MW398649	KP307545	KP307640
*Didymodonasperifolius*	MW398594	JQ890472	KP307600
*Didymodonaustralasiae* (*Trichostomumaustralasiae*)	MW398737	KP307571	KP307651
*Didymodonbrachyphyllus* (*Vinealobryumbrachyphyllum*)	MW398817		
*Didymodonbuckii*	MW398578		
*Didymodoncaboverdeanus*	MW398607		
*Didymodoncalifornicus* (*Vinealobryumcalifornicum*)	MW398819		
*Didymodoncanoae*	MW398584		
*Didymodoncardotii*	MW398729		
*Didymodonchallaensis* (*Trichostomopsischallaensis*)	MW398748		
*Didymodonconstrictus*	MW398613		
*Didymodoncordatus*	MW398664	KP307564	KP307668
*Didymodonditrichoides*	MW398642		
*Didymodoneckeliae* (*Vinealobryumeckeliae*)	MW398826		
*Didymodonedentulus*	MW398685		
*Didymodonepapillatus*	MW398665		
*Didymodonerosodenticulatus*	MW398792	MF536597	MF536635
*Didymodonerosus*	EU835148	MF536609	MF536646
*Didymodonfallax* (*Geheebiafallax*)	MW398779	KP307552	KP307663
*Didymodonferrugineus* (*Geheebiaferruginea*)	MW398796	MF536588	MF536625
*Didymodonfragilicuspis*	KP307482		
*Didymodonfuscus*	MW398689	KP307537	KP307601
Didymodonaff.fuscus		KP307546	KP307615
*Didymodongaochienii*		KP307538	KP307658
*Didymodongelidus*	MW398693		
*Didymodongiganteus*	MW398786	KP307548	KP307669
*Didymodonglaucus*	MW398612		
*Didymodonguangdongensis* (*Vinealobryumguangdongense*)	MW398657		
*Didymodonhedysariformis*	MW398582	KP307569	KP307629
*Didymodonhengduanensis*	MW398629		
*Didymodonhegewaldiorum*	MW398739		
*Didymodonherzogii*	MW398746		
*Didymodonhumboldtii*	MW398667		
*Didymodonicmadophilus*	MW398632	KP307598	KP307604
*Didymodonimbricatus*	MW398646		
*Didymodonincrassatolimbatus*	MW398572		
*Didymodonincurvus*	MW398680		
*Didymodoninsulanus* (*Vinealobryuminsulanum*)	MW398811		
*Didymodonjaponicus*	MW398757		
*Didymodonjimenezii*	MW398622		
*Didymodonjohansenii*	MW398589	KP307542	KP307662
*Didymodonkunlunensis*	MW398610		
*Didymodonlaevigatus*	MW398618		
*Didymodonlainzii*	MW398575		
*Didymodonleskeoides* (*Geheebialeskeoides*)	MW398777	MF536604	MF536642
*Didymodonluehmannii*	MW398718		
*Didymodonluridus*	AY437098	MF536587	MF536624
*Didymodonmaschalogena*	MW398615		
*Didymodonmaximus* (*Geheebiamaxima*)	MW398784	MF536591	MF536628
*Didymodonmesopapillosus*	MW398758		
*Didymodonmolendoides*	MW398687		
*Didymodonmongolicus*	KU058175		
*Didymodonmurrayae*	KP307513	KP307563	KP307650
*Didymodonnevadensis*	MW398730		
*Didymodonnicholsonii* (*Vinealobryumnicholsonii*)	MW398808		
*Didymodonnigrescens*	LC545516	KP307543	KP307611
*Didymodonnorrisii*	MW398830	KP307585	KP307617
*Didymodonnovae-zelandiae*	MW398769		
*Didymodonobtusus*	MW398666		
*Didymodonoccidentalis*		KP307533	KP307599
*Didymodonochyrarum*	MW398763		
*Didymodonparamicola* (*Trichostomopsisparamicola*)	MW398740		
*Didymodonpatagonicus*	MW398675		
*Didymodonperobtusus*	KP307523	KP307539	KP307609
*Didymodonrevolutus* (*Husnotiellarevoluta*)	MW398569	JQ890471	KP307646
Didymodonrevolutusvar.africanus	MW398568		
*Didymodonrigidulus*	MW398602	KP307589	KP307647
Didymodonrigidulusvar.subulatus	MW398672		
*Didymodonrivicola*	MW398599	KP30756	KP307607
*Didymodonsantessoni*	MW398705		
*Didymodonsicculus*	MW398801	MF536606	MF536643
*Didymodonsinuosus*	MW398567	JQ890476	JQ890410
*Didymodonspadiceus* (*Geheebiaspadicea*)	MW398795	MF536593	MF536631
*Didymodonsubandreaeoides*	AY437108	KP307570	KP307630
*Didymodontectorum*	MW398659		
*Didymodontibeticus*	MW398638		
*Didymodontomaculosus*	AY437114		
*Didymodontophaceus*	MW398807	MF536607	MF536644
Didymodontophaceusvar.anatinus		MF536589	MF536626
*Didymodontorquatus*	MW398719		
*Didymodonumbrosus* (*Trichostomopsisumbrosa*)	MW398742		
*Didymodonvalidus*	MW398650		
*Didymodonvinealis* (*Vinealobryumvineale*)	MW398815	JQ890475	KP307606
Didymodonvinealisvar.rubiginosus	MW398822		
*Didymodonvulcanicus*	MW398636		
*Didymodonwaymouthii*	MW398770		
*Didymodonwisselii*	MW398655		
*Didymodonxanthocarpus*	MW398696	KP307534	KP307638
*Didymodonzanderi*	MW398585	KP307535	KP307621
*Dolotortulamniifolia*	MW398555		
*Erythrophyllopsisandina*	MW398546		
*Gertrudiellauncinicoma*	MW398698		
Gertrudiellauncinicomavar.serratopungens	MW398701		
*Guerramontesiamicrodonta*	MW398543		
*Hennediellaheimii*	GQ339750		
*Hennediellapolyseta*	GQ339759		
*Leptodontiumexcelsum*	MW398545		
*Microbryumcurvicolle*		JX679986	JX679936
*Microbryumdavallianum*	MW398557		
*Pseudocrossidiumhornschuchianum*	MW398551	JQ890481	JQ890420
*Pseudocrossidiumrevolutum*	MW398552		
*Pterygoneurumovatum*	MW398560		
*Sagenotortulaquitoensis*	GQ339761		
*Stegonialatifolia*	MW398559		
*Syntrichiaruralis*	MW398564	FJ546412	FJ546412
*Tortulamuralis*	MW398562	JN581679	JQ890421
*Tortulasubulata*	MW398563		
*Triquetrellaarapilensis*	MW398544		
*Tridontiumtasmanicum*	MW398750		

The sequences were aligned by using MAFFT 7.222 (Kazutaka and Daron 2013) and then edited in BioEdit 7.0.1 (Hall 1999). The concatenation of individual *rps*4 and *trn*M-*trn*V fragments was performed by our custom Perl script. The phylogenetic position of the new species was analysed within a comprehensive phylogenetic analysis of *Didymodon* s. lato. species by our previous study (Zhang et al. 2022). Phylogenetic analyses were performed by using the Bayesian Inference (BI) and Maximum Likelihood (ML) methods. Parameter configuration and convergence estimation followed Zhang et al. (2022).

## ﻿Results

**Figure 1.**
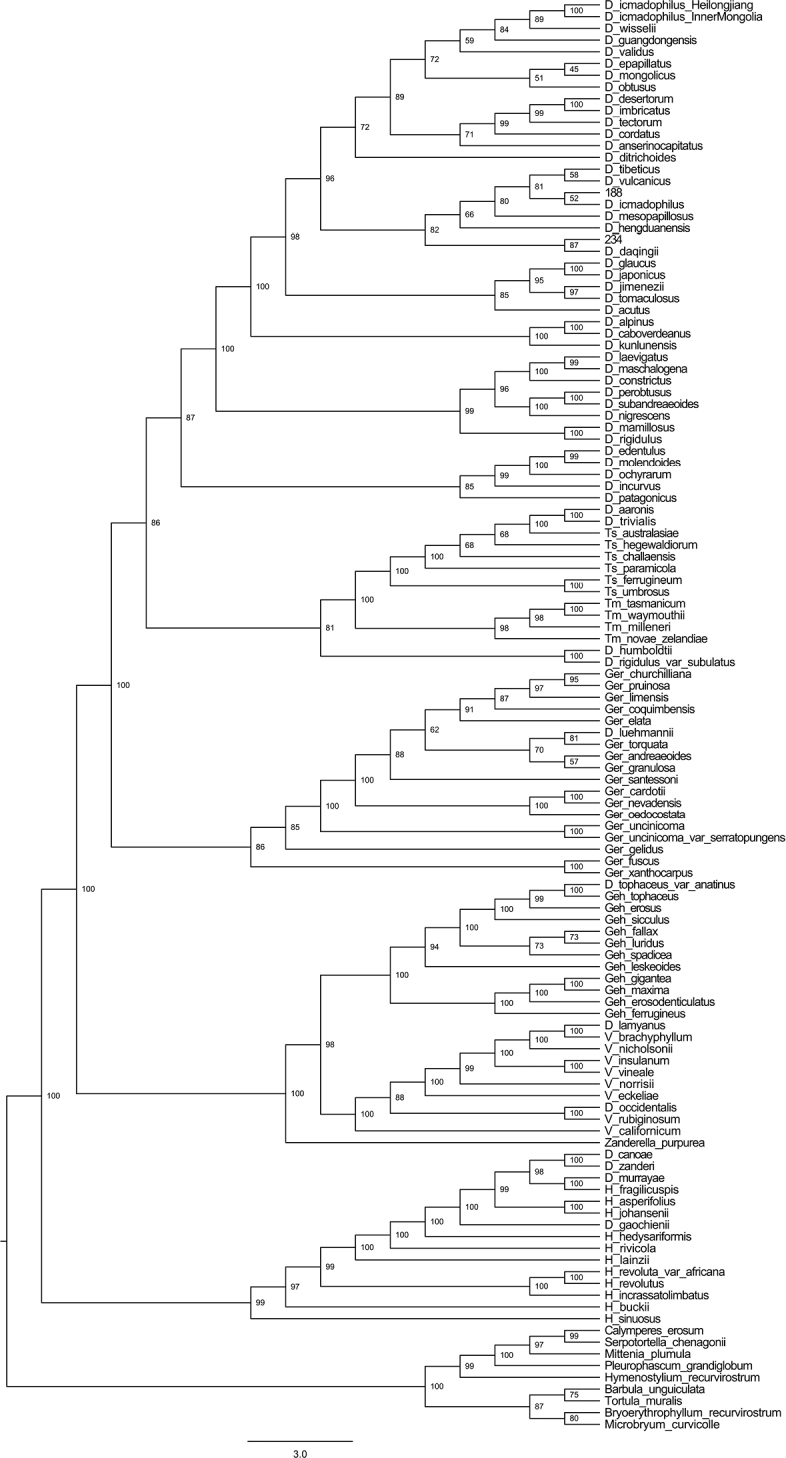
Maximum Likelihood tree inferred from concatenated ITS, *rps*4 and *trn*M-*trn*V datasets. Numbers indicate Maximum Likelihood bootstrap values. The numbers 234 and 188 show the sample of *D.changbaiensis* and an unknown species, respectively.

**Figure 2.**
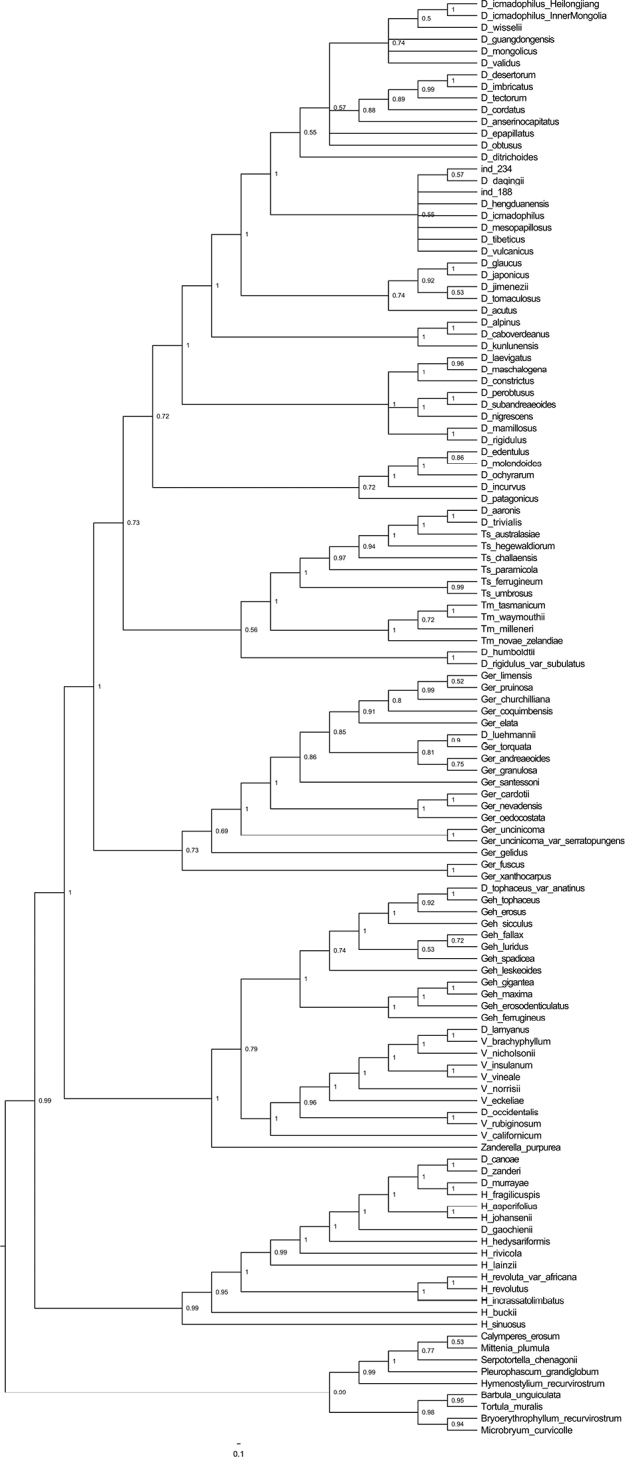
Phylogenetic relationships (50% majority consensus tree) from the Bayesian Inference of the concatenated ITS, *rps*4 and *trn*M-*trn*V datasets. Numbers indicate posterior probability from the BI analysis. The numbers 234 and 188 show the sample of *D.changbaiensis* and an unknown species, respectively.

The combined length of ITS and cpDNA (*rps*4 and *trn*M-*trn*V) is 4622 bp. The position of the new species is different between the BI and ML phylogenetic trees and, thus, both of them are reserved. The topology, based on ML analyses, shows that the new species is sister to *Didymodondaqingii* J. Kou, R.H. Zander & C. Feng and they nested within the monophyletic group comprising *Didymodonhengduanensis* J.A. Jiménez, D.G. Long, Shevock & J. Guerra, *D.icmadophilus* (Schimp. ex Müll. Hal.) K. Saito, *D.mesopapillosus* J. Kou, X.-M. Shao & C. Feng, *D.tibeticus* and *D.vulcanicus* J.A.Jiménez, Hedd. & Frank Müll (Fig. 1). The BI tree was more similar to that of the ML tree, but with weakly-supported values (Fig. 2).

## ﻿Discussion

Our molecular analyses reveal that the new species belongs to *Didymodon* s.str. Morphologically, the combination of characters: concave leaves, plane leaf margins, percurrent to excurrent costa, seldom papillose laminal cells, costa with quadrate or occasionally short-rectangular superficial adaxial cells and absence of costal adaxial stereid band also suggests the placement of the new species in the emended genus *Didymodon* s.str. Zander (1978, 1993, 2013). The new species is distinguished from all congeners by the following combination of diagnostic features: ovate or ovate-lanceolate leaves that are appressed when dry, acute leaf apex, lamina red or reddish-orange with KOH, unistratose lamina throughout, plane and unistratose leaf margins, percurrent costa with one layer of guide cells and without ventral stereids, upper and middle laminal cells with elliptical papillae over the transverse walls between two immediately adjacent cells and basal laminal cells not differentiated from the median cells.

The phylogenetic analyses support that the new species is closely related to *D.daqingii*, a species that was recently described from Inner Mongolia, China (Kou et al. 2019). They have similarity in quadrate or subquadrate ventral cells of costa in the upper middle part of the leaf, bulging costal ventral surface cells, transverse section of costa round at mid-leaf and costa without ventral stereids. However, *D.daqingii* differs from the new species by the long-lanceolate leaves, very fragile and bistratose leaf apices, distally bistratose and recurved leaf margins, costa with guide cells in 2–3 layers, laminal cells with 1–3 simple papillae per cell and yellow-green KOH laminal colour reaction.

There are five species that are related to the new species, based on our phylogenetic analysis. Amongst them, the new species is most similar to *D.tibeticus*, based on morphological characters. They share such distinctive characteristics as the shape of the leaves, lamina red or reddish-orange with KOH, unistratose lamina throughout, plane and unistratose leaf margins and percurrent costa. Nevertheless, *D.tibeticus* can be separated from the new species by the costa with a ventral costal pad of cells and 0–1 layer of ventral stereids, transverse section of costa flattened at mid-leaf and laminal cells with 1–2 simple or bifurcate papillae per cell. Other species, *Didymodonhengduanensis* J.A. Jiménez, D.G. Long, Shevock & J. Guerra, *D.icmadophilus* (Schimp. ex Müll. Hal.) K. Saito, *D.mesopapillosus* J. Kou, X.-M. Shao & C. Feng and *D.vulcanicus* J.A.Jiménez, Hedd. & Frank Müll. are distinguished from the new species by their recurved leaf margins and costa with ventral stereids.

### ﻿Taxonomic treatment

#### ﻿ Didymodon changbaiensis

Taxon classificationPlantaePottialesPottiaceae

J.Kou, C.Feng, H.-X.Xiao & T.-T.Wu sp. nov.

0C56A434-C381-52C7-BCE6-5072940145A6

**Figure 3.**
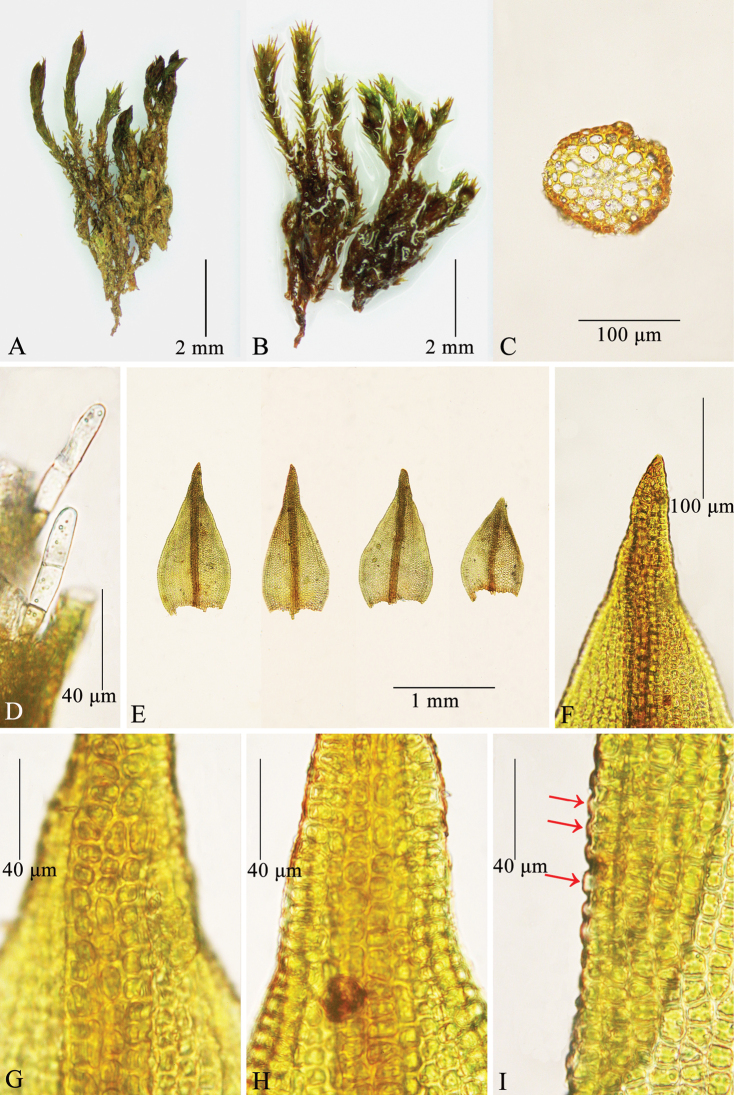
*Didymodonchangbaiensis***A** dry plants **B** moist plants **C** cross-section of stem **D** axillary hairs **E** leaves **F** leaf apex **G** upper part of costa (dorsal) **H** upper part of costa (ventral) **I** upper leaf margin, arrows shows the laminal cells with elliptical papillae over the transverse walls between two immediately adjacent cells. Photographed on 25 May 2022 by Chao Feng from the isotype.

**Figure 4.**
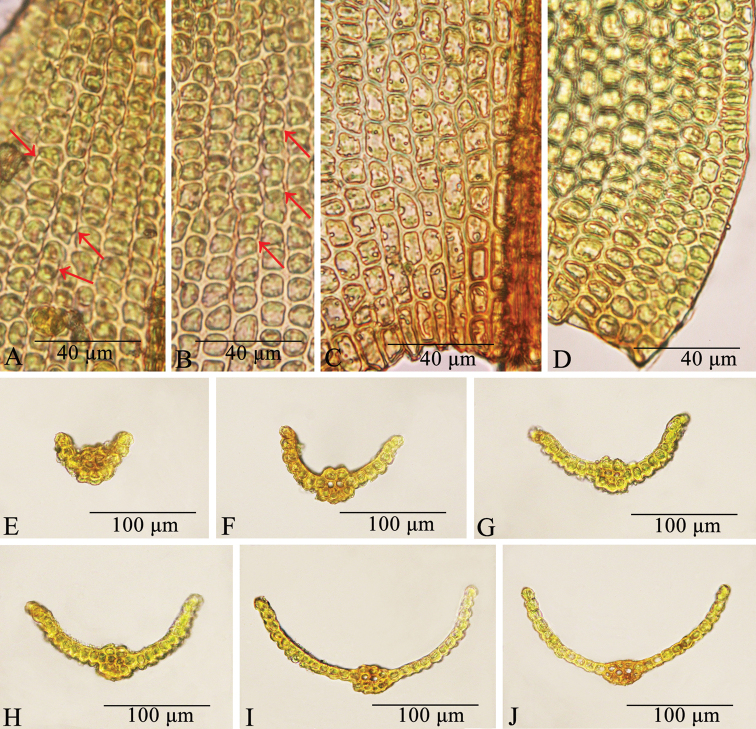
*Didymodonchangbaiensis***A, B** median leaf cells, arrows show the laminal cells with elliptical papillae over the transverse walls between two immediately adjacent cells **C** basal juxtacostal cells **D** basal marginal cells **E–J** cross-sections of leaves, sequentially from apex to base. Photographed on 25 May 2022 by Chao Feng from the isotype.

Figs 3 , 4 Chinese name: 长白山对齿藓

##### Type.

China. Jilin Province: Changbai Mountain, 42°3'35.316"N, 128°3'51.516"E, on soil over rocks, elevation 1864 m, 2 September 2020, *Jin Kou 20200902234* (holotype: NENU!; isotype: NMAC 20200902234!).

##### Diagnosis.

It differs from the otherwise similar *D.tibeticus* in its costa without a ventral costal pad of cells and ventral stereids, transverse section of costa round at mid-leaf and laminal cells with elliptical papillae over the transverse walls between two immediately adjacent cells.

##### Description.

Plants to 1 cm high, growing in dense turfs, brown-reddish below, green above. Stems erect, frequently branched, in transverse section rounded, central strand weakly differentiated, hyalodermis and sclerodermis absent; axillary hairs filiform, usually 3–4 cells long, with one brown basal cell and hyaline upper ones. Rhizoidal tubers absent. Leaves appressed when dry, erect when moist, ovate or ovate-triangular with a broad base, 0.85–1.3 × 0.43–0.65 mm, channelled ventrally in the upper part; lamina completely unistratose, reddish-orange in KOH; apex acuminate to acute, not cucullate; margins entire, plane, completely unistratose; costa 51.7–86.2 µm wide at base, percurrent to short-excurrent; ventral cells of costa in upper middle part of leaf quadrate or subquadrate, sparsely papillose; dorsal cells of costa in upper middle part of leaf quadrate or subquadrate, sparsely papillose; transverse section of costa round at mid-leaf; with 3–4 guide cells in one layer, absence of ventral stereids, 1–2 layers of dorsal stereids, without hydroids, ventral surface cells bulging, not forming a pad of a single layer of cells, papillose, dorsal surface cells papillose; upper and middle laminal cells subquadrate, hexagonal or shortly rectangular, 5.5–11.1 × 8.9–12.2 µm, dorsally with one low elliptical papilla over the transverse walls which reaches the two immediate cells; basal cells weakly differentiated, smooth, basal juxtacostal cells hexagonal or short-rectangular, 11.1–20 × 5.56–10 µm, evenly thick-walled; basal marginal cells oblate, 5.56–10 × 6.67–10 µm, with regular thickened transverse walls and thin longitudinal walls. Gemmae absent. Dioicous. Sporophyte unknown.

##### Etymology.

The specific epithet refers to Changbai Mountain, the type locality.

##### Habitat and distribution.

The new species is currently known only from the type locality at the north slope of Changbai Mountain, Jinlin Province, China, growing on thin soil over rocks.

## Supplementary Material

XML Treatment for Didymodon changbaiensis
